# Mosquito Control Activities during Local Transmission of Zika Virus, Miami-Dade County, Florida, USA, 2016

**DOI:** 10.3201/eid2605.191606

**Published:** 2020-05

**Authors:** Janet C. McAllister, Mario Porcelli, Johana M. Medina, Mark J. Delorey, C. Roxanne Connelly, Marvin S. Godsey, Nicholas A. Panella, Nicole Dzuris, Karen A. Boegler, Joan L. Kenney, Linda Kothera, Lucrecia Vizcaino, Audrey E. Lenhart, John-Paul Mutebi, Chalmers Vasquez

**Affiliations:** Centers for Disease Control and Prevention, Fort Collins, Colorado, USA (J.C. McAllister, M.J. Delorey, M.S. Godsey, N.A. Panella, K.A. Boegler, J.L. Kenney, L. Kothera, J.-P. Mutebi);; Miami-Dade County Mosquito Control Division, Miami, Florida, USA (M. Porcelli, J.M. Medina, C. Vasquez);; Florida Medical Entomology Laboratory, Vero Beach, Florida, USA (C.R. Connelly);; Centers for Disease Control and Prevention, Atlanta, Georgia, USA (N. Dzuris, L. Vizcaino, A.E. Lenhart)

**Keywords:** mosquitoes, Zika virus, Miami-Dade County, Florida, United States, vector-borne infections, mosquito control, flaviviruses, birth defects, viruses, zoonoses

## Abstract

In 2016, four clusters of local mosquitoborne Zika virus transmission were identified in Miami-Dade County, Florida, USA, generating “red zones” (areas into which pregnant women were advised against traveling). The Miami-Dade County Mosquito Control Division initiated intensive control activities, including property inspections, community education, and handheld sprayer applications of larvicides and adulticides. For the first time, the Mosquito Control Division used a combination of areawide ultralow-volume adulticide and low-volume larvicide spraying to effectively control *Aedes aegypti* mosquitoes, the primary Zika virus vector within the county. The number of mosquitoes rapidly decreased, and Zika virus transmission was interrupted within the red zones immediately after the combination of adulticide and larvicide spraying.

Zika virus (ZIKV), a flavivirus that can cause birth defects and is associated with Guillain-Barré syndrome, has rapidly spread throughout the Western Hemisphere (*1*–*3*). The virus is spread primarily by the bite of infected *Aedes aegypti* mosquitoes; sexual transmission and bloodborne transmission also have been documented (*4*,*5*). The southern United States is habitable for *Ae. aegypti* mosquitoes, which are predominantly an urban species. Miami-Dade County, Florida, has well-established *Ae. aegypti* mosquito populations and is a major travel destination (15.8 million visitors reported in 2016 [http://partners.miamiandbeaches.com/tools-and-resources/research-and-statistics]). In addition, the county has a large population of residents who routinely visit countries that had Zika outbreaks in 2016.

In January 2016, the county documented its first travel-associated case of ZIKV infection (*6*). The first cluster of local vector-transmitted cases was identified through epidemiologic investigation in the Wynwood neighborhood of Miami on July 21, 2016, and was confirmed and announced on July 29 (*7*). Following Centers for Disease Control and Prevention (CDC) guidelines (https://www.cdc.gov/zika/public-health-partners/cdc-zika-interim-response-plan.html) a “red zone” or travel warning was declared for pregnant women to avoid unnecessary travel to areas within ≈1 square mile around the cluster of cases (Figure 1). Subsequent clusters were identified south of 28th Street in Miami Beach on August 19, initiating a second red zone of 1.5 square miles. On September 16, a cluster was identified north of 28th Street in Miami Beach, expanding the second red zone by another 1.5 square miles to the north. On October 13, a fourth cluster was identified, and a red zone was declared in the Little River area of Miami, although all but 1 case occurred before October 13.

**Figure 1:**
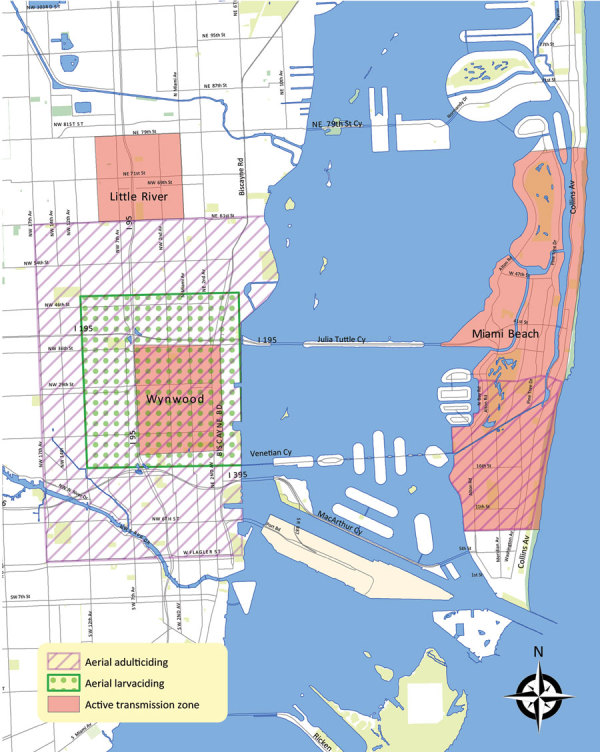
Locations of declared zones where clusters of locally acquired vectorborne Zika virus transmission were identified and aerial mosquito control activities conducted, Miami-Dade County, Florida, USA, 2016.

As each cluster was identified, Miami-Dade County Health Department began intensified epidemiologic surveillance to detect additional cases. Concurrently, the Miami-Dade County Mosquito Control Division (MCD) initiated intensive mosquito control activities within each new cluster of local transmission. We describe the mosquito control activities used to address the clusters of locally acquired ZIKV and their effect on subsequent mosquito numbers and Zika transmission.

## Mosquito Control Methods

The Miami-Dade County Health Department notified the county MCD of all suspected or confirmed ZIKV infections. Relevant addresses (i.e., home, work) associated with each notification were inspected for the presence of *Ae. aegypti* mosquitoes. On the basis of the inspection, source reduction and application of larvicide, adulticide, or both were performed as needed. In addition, MCD attempted to inspect all properties in a 150-meter radius of the case-patient’s house. MCD made multiple visits to reach all homes. At a minimum, front yards of all properties were evaluated, and when house occupants granted permission, backyards as well. MCD left educational materials at all properties.

In the red zones, control activities expanded to include all properties within the zone. Inspection of individual properties helped the MCD define the most common containers that served as larval habitats. The MCD recorded only presence or absence of larvae and did not attempt to quantify or identify the species; thus, mosquito species other than *Ae. aegypti* might have been present. Regardless of the mosquito species present, the MCD treated them either by removing the water (dumping) or applying a larvicide. During July 23–December 29, MCD conducted 352,209 property inspections countywide. The Wynwood red zone had 1,721 parcels on which 5,974 inspections occurred. During August 19–December 29, MCD conducted 8,755 inspections in the southern Miami Beach (1,980 parcels) and 6,872 inspections in the northern Miami Beach (2,783 parcels) red zones. In Little River, MCD conducted 3,239 inspections on the 2,075 parcels within the red zone during October 14–December 29. 

The 24,795 inspections in the 4 red zones identified a total of 2,720 containers with larval mosquitoes. Most (92%) containers with larval mosquitoes were of 3 types: drains, predominately storm drains (33%); plants, predominately bromeliads (35%); and small containers that were easily dumped (25%). Saucers beneath potted plants were included in the small containers–dumpable category. The next most common larval mosquito habitat was tires, constituting 4% of larvae-positive containers. The remaining container types represented <1% of the total: small containers–permanent, plastic construction barriers, fountains, pools, boats, ponds, ditches, and hot tubs. 

**Figure 2:**
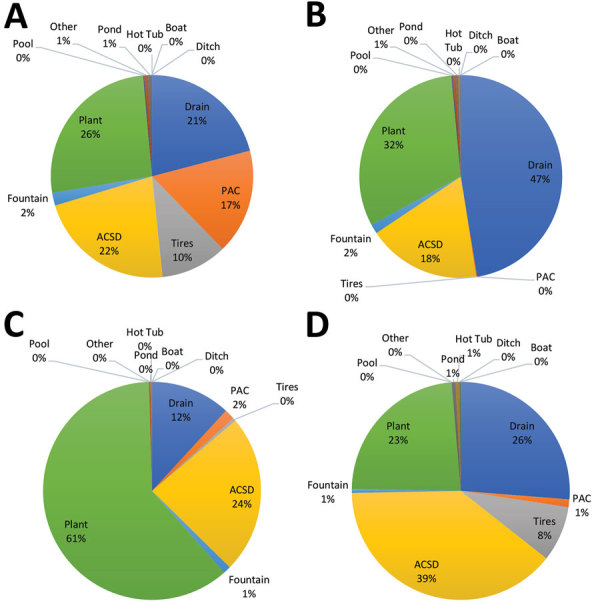
Relative abundance of container types with larval *Aedes aegypti* mosquitoes, Miami-Dade County, Florida, USA, 2016. A) Wynwood; B) southern Miami Beach; C) northern Miami Beach; D) Little River. PAC, permanent artificial container; ACSD, artificial container/small–dumpable.

The distribution of the most common container types was not uniform across the county. In Wynwood, plants were the most abundant container with larvae (26%) (Figure 2, panel A). In northern Miami Beach, plants accounted for 61% of the containers with larvae (Figure 2, panel B). In southern Miami Beach, drains contributed almost half (47%) of the larval sites (Figure 2, panel C). In Little River, small containers–dumpable accounted for 39% of containers with mosquito larvae (Figure 2, panel D). In addition, red zones received ultralow-volume (ULV) spraying of adulticide and low-volume spraying of larvicide delivered by airplane or truck-mounted equipment (Table 1).

**Table 1:** Mosquito control products used by the county Mosquito Control program, Miami-Dade County Florida, USA, 2016

Product name	Active Ingredient	Life stage targeted	Method of application	Application rate
Biomist*	Permethrin/piperonyl butoxide	Adult	Backpack/truck	0.0035 lb/acre
Duet*	Sumithrin and prallethrin	Adult	Backpack/truck	0.0035 lb/acre
Zenivex†	Etofenprox	Adult	Truck	0.0035 lb/acre
DeltaGard‡	Deltamethrin	Adult	Truck	0.0035 lb/acre and 0.007 lb/acre
Dibrom§	Naled	Adult	Airplane	0.1 lb/acre
Vectobac WGD¶	*Bacillus thuringiensis israelensis*	Larva	Truck/airplane/hand	0.5 lb/acre
??Abate#	Temephos	Larva	Backpack/hand	**
Altosid†	Methoprene	Larva	Hand	**

*Clarke, https://www.clarke.com. †Central Life Sciences, https://www.centrallifesciences.com. ‡Bayer CropScience LP, https://www.bayer.com. Rate of application was increased to the maximum amount allowable by the label after field trials to determine effective rate were concluded October 10, 2016. §AMVAC, https://www.amvac.com. ¶Valent Biosciences, https://www.valentbiosciences.com #BASF, https://agriculture.basf.com. **Application rate depended on the container.

## Mosquito Surveillance

A routine surveillance system for *Ae. aegypti* mosquitoes was not in place before August 2016. In each red zone, surveillance for adult *Ae. aegypti* mosquitoes was initiated as soon as a new zone was identified. BG Sentinel traps enhanced with BG-lures (BioGents, https://eu.biogents.com) and dry ice were deployed. Trap density was 17–19 traps/zone/night. Adult mosquitoes from each trap were counted and identified daily until the red zone designation was removed. The predominant species collected in the BG Sentinel traps was *Ae. aegypti* (86%), followed by *Culex quinquefasciatus* (L.) (14%). All other mosquito species comprised <1%. To compare different treatment strategies in Wynwood, we set additional traps in an area around the red zone that received aerial adulticide applications only and inside the red zone where both aerial adulticide and larvicide were applied. Because traps were not readily available in August and early September, traps were moved after 2 weeks from the Wynwood adulticide only area for use in subsequent red zones. As a result, continued surveillance in the area that received aerial adulticide only was not available for longer-term (6 weeks) comparison to aerial adulticide plus larvicide treatments.

## Insecticide Resistance

### Laboratory Assays

The insecticide resistance status of *Ae. aegypti* mosquitoes in Miami-Dade County was not known at the beginning of the outbreak. At the same time that the intensified mosquito control activities began in Wynwood and Miami Beach, *Ae. aegypti* eggs and adults were collected to evaluate their susceptibility to the active ingredients found in various commercial adulticide products, including those routinely used by the MCD. Eggs were reared in an insectary at 27°C and 80%–90% humidity, with 14 h daylight, and the resulting adults were used in the laboratory bioassays. CDC bottle bioassay (*8*) was performed using technical-grade permethrin, 43 μg/bottle; deltamethrin, 0.75 μg/bottle; etofenprox, 12.5 μg/bottle; sumithrin, 20 μg/bottle; naled, 2.25 μg/bottle; and malathion, 400 μg/bottle. Bottle concentrations and threshold times were based on prior calibration of the assay as described previously (*8*). All technical-grade insecticides came from ChemService Inc. (https://www.chemservice.com). *Ae. aegypti* Orlando strain mosquitoes were used as a susceptible comparison colony. This colony was started in 1952 at what is now the US Department of Agriculture’s Agricultural Research Service, Center for Medical, Agricultural and Veterinary Entomology (Gainesville, FL, USA). The CDC bottle bioassay revealed high levels of resistance to all synthetic pyrethroids at the diagnostic time; sumithrin (3%–14% death), etofenprox (1%–7% death), permethrin (2%–12% death), and deltamethrin (5%–65% death). We found no resistance to malathion or naled (Table 2).

**Table 2:** Percentages of mosquito populations susceptible to active ingredients or products used for adult mosquito control in laboratory bioassays and field tests of *Aedes aegypti* mosquitoes, Miami-Dade County, Florida, USA, 2016*

Chemical/product	Bottle dosage, μg/bottle	Mosquito death, %		
		CDC bottle bioassay	At 1/2 label rate in field assay	At full label rate in field assay
Naled†	2.25	100	NA	NA
Malathion†	400	100	NA	NA
Deltamethrin/DeltaGard‡	0.75	5–65	80	93
Etofenprox/Zenivex§	12.5	1–7	19	57
Permethrin/Biomist¶	43	2–12	33	NA
Sumithrin/Duet¶	20	3–14	44	NA

*NA, test not conducted because mosquitoes were susceptible to active ingredient or field test results excluded it from further testing. †Used in bottle bioassays only. No field tests were conducted because mosquitoes were susceptible to this chemical. ‡Bayer CropScience LP, https://www.bayer.com. §Wellmark International, https://www.bpia.org. ¶Clarke, https://www.clarke.com.

### Field Assays

Because resistance in laboratory assays does not directly translate to product failure in the field, we field-tested commercial products to find the most efficacious pyrethroid product for use in truck-mounted ULV spraying. The MCD used the midlabel rate (Table 1) for product application before and early in the outbreak. Mosquitoes collected in BG sentinel traps were held in a BugDorm2 Insect Tent (BioQuip, https://www.bioquip.com) and supplied with 10% sucrose until use in field testing. Field testing consisted of placing adult mosquitoes in cages, then exposing them to the commercial product applied at the mid-label rate with a truck-mounted Grizzly ULV Sprayer (Clarke, https://www.clarke.com). Cages were 7.6 m and 15.2 m from the road. Fifteen minutes after insecticide exposure, mosquitoes were transferred to clean holding containers (236.5-mL cardboard ice cream cups covered with netting) and given access to a 10% sucrose solution. Deaths were recorded 24 h after treatment (Table 2). Additional field testing was conducted using the highest label rate (Table 1) of DeltaGard (deltamethrin; Bayer CropScience LP, https://www.environmentalscience.bayer.com) and Zenivex (etofenprox; Central Life Sciences, https://www.centralmosquitocontrol.com). We chose Zenivex for additional testing because the wind appeared to shift direction during the initial application, causing the treatment to not fully reach all cages. We chose DeltaGard because it performed the best in both bottle bioassay and the previous field testing using the midlabel rate. DeltaGard was selected as the best performing product when applied at the highest label rate (0.00134 lb/acre) and was used for truck-mounted spraying in the northern Miami Beach and Little River red zones where aerial spraying did not occur.

## Insecticide Treatments

In addition to treating vegetation with a synthetic pyrethroid during property inspections, MCD applied adulticides using trucks throughout the county using pyrethroid products (Table 1). Biomist 30+30 (Clarke) was the most commonly used product before insecticide resistance testing. Because mosquito numbers remained high after initial hand- and truck-based applications, the MCD opted to include aerial applications of Dibrom (AMVAC, https://www.amvac.com), a product containing naled. The red zones of Wynwood and southern Miami Beach received 4 aerial applications of Dibrom. The first 2 applications occurred within 4 days of each other, followed by 2 more applications at 1-week intervals. A 10 square mile area centered on the red zone in Wynwood and a 1.5-mile area encompassing the southern part of Miami Beach were treated. Because of reduced daylight during fall and the start of the school year, aerial applications in the subsequent red zones were not feasible. In addition, insecticide field testing showed that the highest label rate of DeltaGard could be effective (93% death). Thus, in the red zones in northern Miami Beach and Little River, ≈1.5 square mile each were treated with DeltaGard using a truck-mounted Grizzly ULV Sprayer. Treatments occurred on a similar schedule as was used for aerial spraying, with the initial 2 treatments within 4 days of each other followed by 2 more treatments at weekly intervals.

Vectobac WGD (Valent Biosciences, https://www.valentbiosciences.com), a larvicide product containing *Bacillus thuringiensis israelensis*, was applied every 7 days for 4 weeks in all 4 red zones. This larvicide was applied by aircraft in Wynwood and by a CSM3 Turbine Vector Sprayer/Duster (Buffalo Turbine, https://buffaloturbine.com) mounted on a truck in southern Miami Beach, northern Miami Beach, and Little River. In Wynwood, a 2 m^2^ area was treated, but in the other red zones, larvicide treatments covered the same areas as the adulticide treatments.

## Effect on Mosquito Abundance and Zika Infections

Because the response to the Zika outbreak in southern Florida was an emergency public health intervention, there was no time to set up proper controls. Therefore, we cannot evaluate properly using common comparison techniques the effect of the interventions. Instead, we used a changepoint analysis. A changepoint occurs if a time at which the statistical properties of the ordered sequence of observed case counts change. Case counts evaluated here are adult *Ae. aegypti* counts from BG Sentinel traps. A sequence can have >1 changepoint. In this analysis, the characteristic we assessed is the mean *Ae. aegypti* count change during the time observed. We consider 2 hypotheses: 1) *Ae. aegypti* counts during the entire period derive from a Poisson distribution with a constant mean, and 2) >2 time intervals exist, in each of which the *Ae. aegypti* counts derive from Poisson distributions with different means. We used a likelihood approach using binary segmentation, as described previously (*9*), and implemented in the R package changepoint (*10*) to identify whether the data were consistent with hypothesis 1 or hypothesis 2. With each binary segmentation of the sequence, the Akaike information criterion with a correction for small sample size (AICcs) of the models with and without a changepoint, were computed. A model with the changepoint was considered a better fit if its AICc was smaller by >6 than the AICc of the model without the changepoint, which corresponds roughly with a type I error of 0.05.

We obtained dates of human cases from the Florida Deptartment of Health website (http://www.floridahealth.gov/newsroom/2016/11/113016-zika-update.html). Because the Little River red zone was declared when mosquito numbers were naturally dropping due to seasonality and mosquitoborne transmission had ceased by the time that red zone was identified, we did not evaluate changepoint analysis and transmission after spraying.

**Figure 3:**
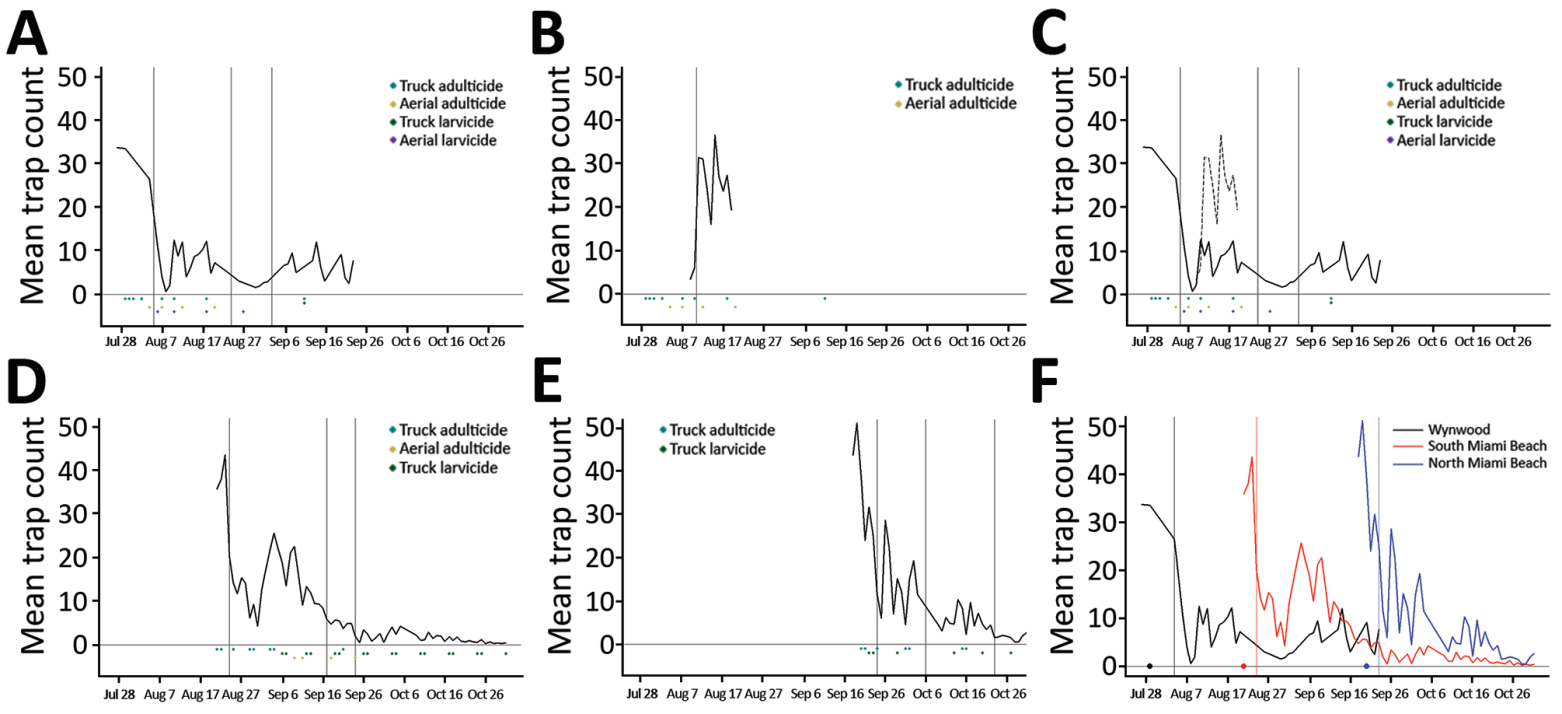
Changepoint in mean counts of *Aedes aegypti* mosquitoes from areas receiving adulticides and larvicides, Miami-Dade County, Florida, USA, 2016. Vertical lines indicate dates of changepoints for mean *Ae. aegypti* counts. A) Wynwood neighborhood; B) 10-mile region around the Wynwood neighborhood; C) combined Wynwood neighborhood (solid line) and 10-mile region around the Wynwood neighborhood (dotted line); D) southern Miami Beach; E) northern Miami Beach; F) Wynwood and Miami Beach combined. Points on the horizontal axis represent the first day of insecticide spraying; vertical lines show the first changepoint.

Although we cannot make a statistical association with the location of the changepoints in the other red zones, it is interesting that the first changepoints occurred after adulticide treatments began. In the Wynwood red zone that received both aerial adulticide and larvicide (Figure 3, panel A), this changepoint represents a large drop in mean *Ae. aegypti* counts. With further adulticiding and larviciding, the counts remained low and, in fact, dropped further on August 24. On approximately this date, insecticide applications stopped, and this date is followed by a third changepoint at which mean *Ae. aegypti* counts increased again.

**Figure 4:**
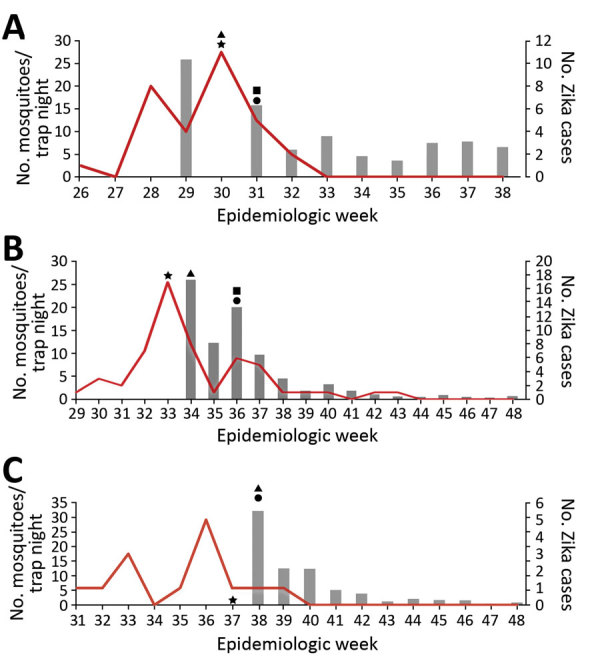
Average number of *Aedes aegypti* mosquitoes and locally acquired Zika virus cases by epidemiologic week during the period of insecticide application, Miami-Dade County, Florida, USA, August–November 2016. A) Wynwood; B) southern Miami Beach; C) northern Miami Beach. Gray bars indicate mosquito counts; red line indicates Zika cases. Star indicates week cluster of locally acquired cases identified; square indicates first aerial adulticide application; triangle indicates first truck adulticide application; circle indicates first areawide (truck or aircraft) larvicide application. Cases are reported by date of symptom onset or date of specimen collection if no symptoms were present. Actual infection occurred before reporting date and is typically >1 week before the reporting date.

The only changepoint in the 10-mile area around Wynwood that received adulticide only occurred after the adulticiding began. We do not know what the mean *Ae. aegypti* counts were before August 9 (Figure 3, panel B). However, superimposing the counts for 10-mile region around the Wynwood neighborhood over those for the Wynwood neighborhood (as shown in Figure 3, panel C) showed that the mean *Ae. aegypti* counts for August 9 were comparable. Counts before August 9 might also have been comparable, but we have no way to verify that possibility. *Ae. aegypti* counts then increased in the region around Wynwood, whereas mean counts within Wynwood remained low. One possible explanation for this increase is that larviciding was not done in the 10-mile region around Wynwood. This observation reinforces the concept that both adulticiding and larviciding are needed to quickly reduce mosquito populations and maintain suppression. As reported previously (*7*), detection of new Zika virus infections in Wynwood stopped after adulticide treatments began (Figure 4).

In the southern Miami Beach red zone, we again saw that the first changepoint occurred after the first adulticide treatments (Figure 3, panel D). There was a slight increase in mosquito count after the initial decrease (although this is not statistically a changepoint). Once the larvicide treatments began on September 6, mean *Ae. aegypti* counts decreased again, and 2 changepoints in mean counts followed the start of the larviciding. New cases of Zika virus ceased immediately after the first aerial adulticide treatments. However, for 4 weeks, single cases occurred roughly weekly after the last aerial adulticide treatment.

In the northern Miami Beach red zone, the first changepoint again occurred after the first adulticide treatments (Figure 3, panel E). After larviciding, the mean counts remained low and were followed by 2 more changepoints in mean *Ae. aegypti* counts. Again, for a third time no new Zika virus infections occurred after the first adulticide treatments.

We do not know what the *Ae. aegypti* counts were ahead of treatments or what would have occurred if treatments had not been initiated. However, graphing the *Ae. aegypti* counts from Wynwood and Miami Beach together (Figure 3, panel F) suggests that, before treatments began, the mosquito counts remained consistently high throughout the season (≈30–50 mosquitoes per trap). Only after the first adulticiding in each area did the mean mosquito counts drop statistically and vector-transmitted Zika virus infections cease.

## Lessons Learned

The purpose of using adulticides in an outbreak is to immediately reduce the number of adult mosquitoes (particularly older ones) that might be capable of transmitting disease. We observed interruption of vectorborne Zika virus transmission in Wynwood and both red zones in Miami Beach after beginning intensive adulticiding. In the United States, adulticide treatments using space-spraying techniques against *Ae. aegypti* mosquitoes have been shown to quickly knock down adult populations (*11*). However, these adult mosquito reductions are transient because not all mosquitoes will be active (and thus exposed) during application; in addition, adulticides do not control larvae and pupae, new adult mosquitoes will quickly repopulate an area. Therefore, repeated adulticide treatments are needed to eliminate newly emerging mosquitoes.

The use of larvicides alone does not immediately control adult mosquito populations, and it is not unusual to see the effect of larvicides until several weeks after their application (*12*). Our observation in Wynwood, where mosquito numbers remained suppressed when both adulticide and larvicide applications occurred, compared with the area that received only adulticide treatments, reinforces the necessity of a combination approach to achieve and sustain impact. Observations that aerial adulticiding and combinations of adult and larval mosquito control can successfully interrupt vectorborne disease transmission have been previously reported. Aerial adulticiding in California stopped West Nile virus transmission in an area that received the treatment, whereas cases continued to occur in untreated surrounding areas (*13*). Although larvicides are typically not recommended as part of a malaria control program, an example of the effect of both adulticide and larvicide contributing to reduction of disease was documented in Kenya, where transmission of malaria decreased substantially after a combination of larvicide and insecticide-treated nets were used (*14*). The combination approach can prolong the recovery of a treated mosquito population because adult mosquitoes are killed, thereby immediately interrupting virus transmission and deposition of new eggs, and emergence of new adults is interrupted by the larvicide, keeping the population from quickly rebounding and thus preventing ongoing virus transmission.
